# Protecting role of cosolvents in protein denaturation by SDS: a structural study

**DOI:** 10.1186/1472-6807-8-29

**Published:** 2008-06-03

**Authors:** Catherine Michaux, Jenny Pouyez, Johan Wouters, Gilbert G Privé

**Affiliations:** 1 Chemistry department, CBS lab, CPTS group, 61 rue de Bruxelles, B-5000 Namur, Belgium; 2 Division of Cancer Genomics and Proteomics, Ontario Cancer Institute 101 College Street, Toronto, Ontario, M5G 1L7, Canada

## Abstract

**Background:**

Recently, we reported a unique approach to preserve the activity of some proteins in the presence of the denaturing agent, Sodium Dodecyl Sulfate (SDS). This was made possible by addition of the amphipathic solvent 2,4-Methyl-2-PentaneDiol (MPD), used as protecting but also as refolding agent for these proteins. Although the persistence of the protein activity in the SDS/MPD mixture was clearly established, preservation of their structure was only speculative until now.

**Results:**

In this paper, a detailed X-ray study addresses the pending question. Crystals of hen egg-white lysozyme were grown for the first time in the presence of MPD and denaturing concentrations of SDS. Depending on crystallization conditions, tetragonal crystals in complex with either SDS or MPD were collected. The conformation of both structures was very similar to the native lysozyme and the obtained complexes of SDS-lysozyme and MPD-lysozyme give some insights in the interplay of protein-SDS and protein-MPD interactions.

**Conclusion:**

This study clearly established the preservation of the enzyme structure in a SDS/MPD mixture. It is hypothesized that high concentrations of MPD would change the properties of SDS and lower or avoid interactions between the denaturant and the protein. These structural data therefore support the hypothesis that MPD avoids disruption of the enzyme structure by SDS and can protect proteins from SDS denaturation.

## Background

Sodium dodecyl sulfate (SDS) is a highly effective and widely used protein denaturant [1,2]. Our previous work has shown that amphipathic solvents, like 2-methyl-2,4-pentanediol (MPD), a commonly used precipitant for crystallization studies [3], can protect proteins from SDS denaturation, and in several cases can drive the transition from the SDS-denatured state to a functional folded state [4]. This protecting effect of MPD is observed with a wide range of proteins including membrane proteins and soluble enzymes, and is not applicable if proteins are denatured in guanidine or urea, two other denaturant agents. In the case of hen egg-white lysozyme, SDS concentrations above 1.0 mM abolished the activity of the enzyme in the absence of MPD. However, in 2 M MPD, the activity was preserved in the presence of SDS. In addition, when the enzyme was first denatured with SDS for 24 hours prior to adding MPD, full enzymatic activity was recovered in 2 M MPD following a further 24 hour incubation period. Although the persistence of the enzyme activity in the SDS/MPD mixture was clearly established, preservation of its structure was only speculative until now.

In the present contribution, a detailed X-ray study addresses the pending question. Crystals of hen egg-white lysozyme could be grown for the first time in a SDS/MPD medium, providing support that adding MPD to proteins can avoid SDS denaturation. The obtained complexes of SDS-lysozyme and MPD-lysozyme give some insights in the interplay of protein-SDS and protein-MPD interactions. A previous report described the structure of cross-linked lysozyme crystals soaked in SDS solutions [5]. In the latter, unlike both structures described in this paper, SDS was found at three different locations inside the protein inducing a major structural deformation in the interior of lysozyme.

## Results and discussion

### Crystallization

Crystals of hen egg-white lysozyme solubilized in 4 mM SDS, a usually denaturing condition, and 2 M MPD were obtained, without any cross-linking, contrary to the study of Yonath et al. [5], in two different conditions (see Materials and Methods). Tetragonal crystals, called "form I" crystals, were obtained from a classical sodium acetate solution at pH 4.6. In this condition, few MPD, slightly volatile, is expected to remain in the drop after equilibration of the protein droplet with the MPD-free reservoir solution (Table 1). To enhance the concentration of SDS and MPD into the drop, and taking into account results from the literature [6], crystals were also obtained by equilibrating protein solutions with reservoir solutions of 50 mM Tris pH8, 70% MPD and 6 mM SDS resulting in "form II" crystals. Both crystals belong to the same tetragonal space group, P4_3_2_1_2. Form I crystals diffracted X-rays to 2.3 and form II diffracted to 1.75Å.

**Table 1:** Statistics of data collection and structure refinement

	**Form I**	**Form II**
**Expected MPD concentration in the drop after equilibration**	close to 0 M	>4 M
**Expected SDS concentration in the protein droplet**	~2 mM	~5 mM

**Data collection**		

**Space group**	P4_3_2_1_2	P4_3_2_1_2
**Unit-cell parameters (Ǻ)**	a = b = 77.59, c = 37.57	a = b = 77.90, c = 37.53
**Maximum resolution (Ǻ)**	2.3	1.75
**Unique reflections**	5468	12247
**Redundancy**	8.1	10.1
**Completeness (%)**	99.7(100)	99.7(99.9)
**Avg. I/σ**	21.9(8.5)	18.7(3.2)
**R_merge _(%)**	8.22(22.72)	4.5(20.2)

**Refinement statistics**		

**R (%)^a^**	20.18	17.03
**R_free _(%)^a^**	21.40	20.84
**RMSD bond lengths (Ǻ)**	0.021	0.066
**RMSD angle distances (Ǻ) RMSD bond angles (°)**	1.92	0.061
**Average B value (Ǻ^2^)**		
**Protein atoms**	14.91	14.63
**MPD molecules**	/	53.47
**SDS molecule**	45.94	/
**Water molecules**	20.66	26.64
**Ramachandran plot** Most favored, additional, generously allowed (%)	85.0/13.3/1.8	89.4/10.6/0.0
**No. of MPD molecules**	0	2
**No. of SDS molecules**	1	0
**No. of Na ion**	0	1
**No. of Cl ion**	0	2
**No. of water molecules**	69	118

Values listed in parentheses are for the highest resolution.

^a ^R_factor _= ∑||*F*_*o*_| - |*F*_*c*_||/∑|*F*_*o*_|. R_free _was calculated with 5% of the reflections set aside randomly throughout the refinement.

### Structure overview

**Figure 1:**
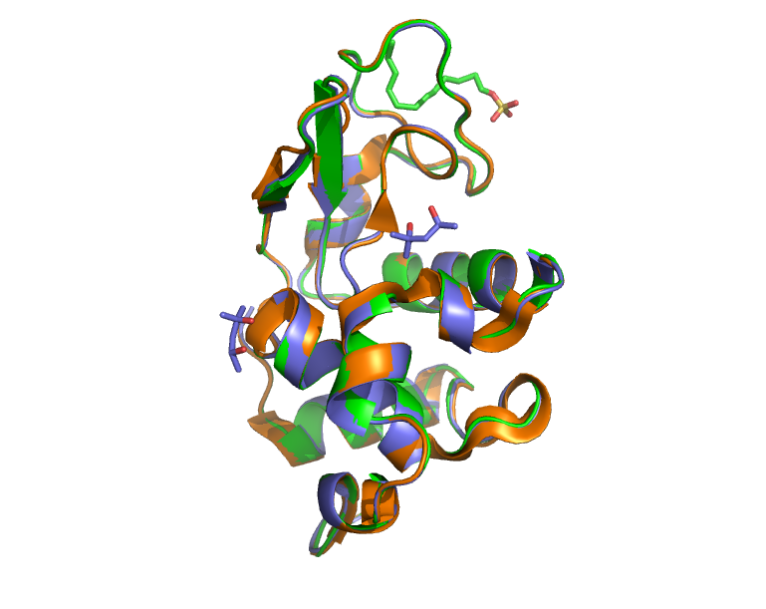
Superimposition of crystal structure of lyzozyme in the native state (1Z55; orange), co-crystallized with SDS (form I; green) and with SDS/MPD (form II; blue).

Unlike the one obtained by Yonath et al [5], the overall conformation of both structures is very similar to native lysozyme (PDB code 1Z55). The RMSD values calculated using the C_α _atoms, after superimposition with the native structure, are 0.27 and 0.20 Å for form I and form II crystals, respectively (Fig. 1). No large structural changes are observed and the interior of the enzyme is intact. The most significant variations are observed at the surface of the proteins in loops where electron densities are less well-defined (for flexible residues like arginine and lysine). These differences are probably due to uncertainties from the model or intrinsic thermal behavior.

The presence of high concentration of MPD can therefore avoid disruption of the structure of the enzyme by SDS. Indeed, as shown by Yonath [5], SDS, by binding deeply into the hydrophobic core of the protein, induces separation of the two wings of lysozyme. In addition, the form I crystal, where few MPD is expected to remain in the drop, shows that MPD was able to induce irreversibly the correct folding of lysozyme in the presence of a denaturing concentration of SDS (2 mM).

### Interaction of SDS or MPD with lysozyme

**Figure 2:**
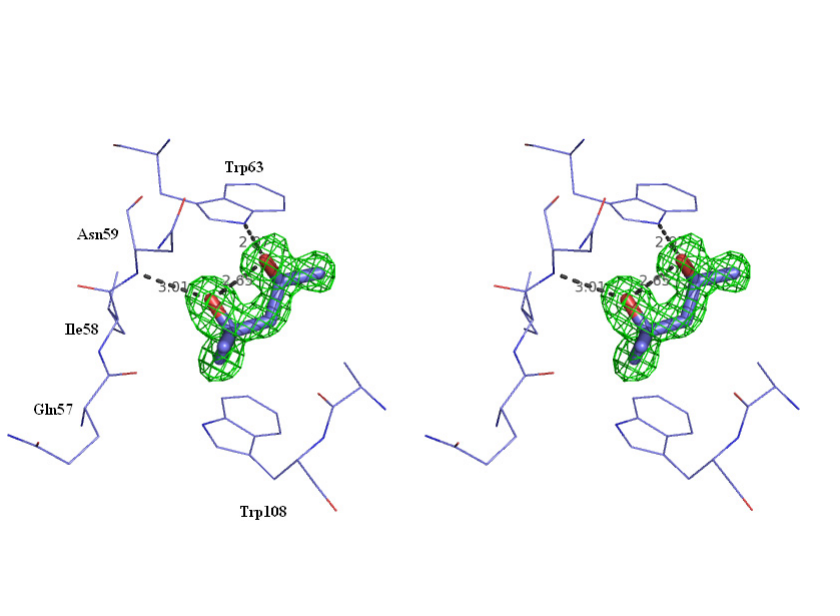
**Stereoview of the complex between lysozyme and MPD in the C-subsite.** Electron density of the *2F*_*O*_-*F*_*C *_map is contoured at 1σ level for MPD.

**Figure 3:**
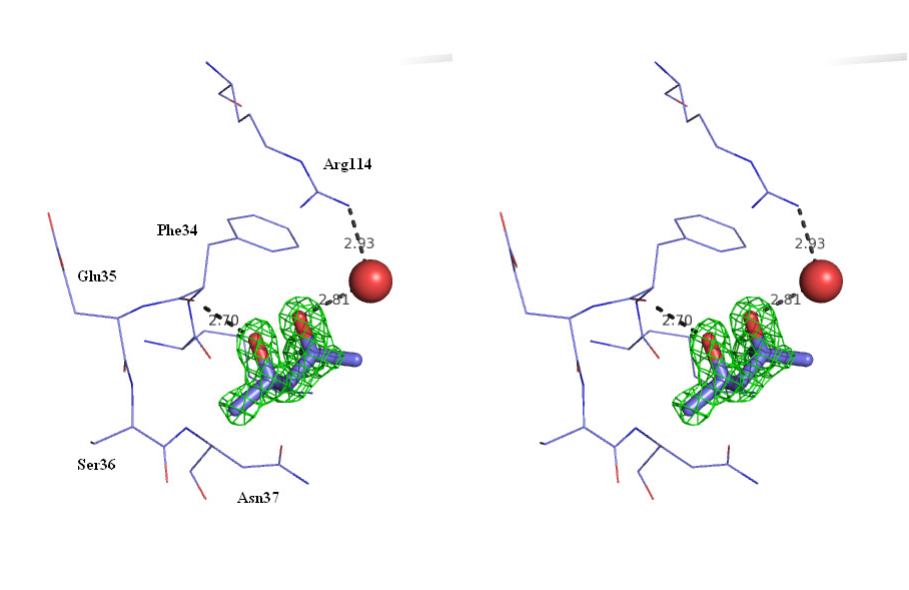
**Stereoview of the complex between lysozyme and MPD on the surface of the protein.** Electron density of the *2F*_*O*_-*F*_*C *_map is contoured at 1σ level for MPD.

Depending on the used reservoir solution, different small molecules were found co-crystallized with lysozyme (Table 1). SDS and MPD molecules could be identified unambiguously in the density maps due to their characteristic shape. With condition II buffer, a first MPD molecule, in the S configuration, occupies the C-subsite in the sugar-binding cleft of the protein formed by residues Asn59, Trp63, Ile58, Ala107 and Trp108 (Fig. 2). This was also found to be the binding site of alcohol molecules like ethanol, propanol, butanol and pentanol [7] and of denaturants, used at non-denaturing concentrations, like DMSO, guanidinium hydrochloride [8] and urea [9,10]. This is in agreement with the assumption that generally MPD molecules prefer to bind to hydrophobic site and MPD binding is penetrative, leading to displacement of water molecules in grooves and cavities [3]. The hydrophobic part of the molecule interacts with Trp108 (contact ≤ 3.5Ǻ) and both alcohol groups are H bonded to the backbone of Asn59 and to Trp63, respectively. A second MPD molecule, in the S configuration, is bound on the surface between two lysozyme molecules, close to Arg114 (Fig. 3). No molecules of SDS were observed in the electronic density map.

The S configuration of both MPD molecules is quite clear by lowering the contour level to around 0.6 but at this stage, it is impossible to deduce that S-MPD is more stabilizing than the R one.

A comparison of the above structure with the one of lysozyme crystallized in the presence of MPD, without SDS, (PDB code 1DPW, 1.64 Ǻ) [6] yields RMSD of 0.14 Ǻ for the superposition of C_α _atoms. The location of the second MPD molecule in the present study matches the one previously described, except the configuration which is switched. The water molecule between MPD and Arg114 is also conserved. Moreover, two chloride ions were identified in the same binding site as in the 1DPW structure. Additionally, a sodium ion was observed ligated to the main chain carbonyl oxygen's of residues Ser60, Cys64, Arg73, Oγ of Ser72 side chain, and two water molecules. This sodium ion binding site was present in the enzyme in the presence of low concentration of DMSO and guanidinium chloride [8].

**Figure 4:**
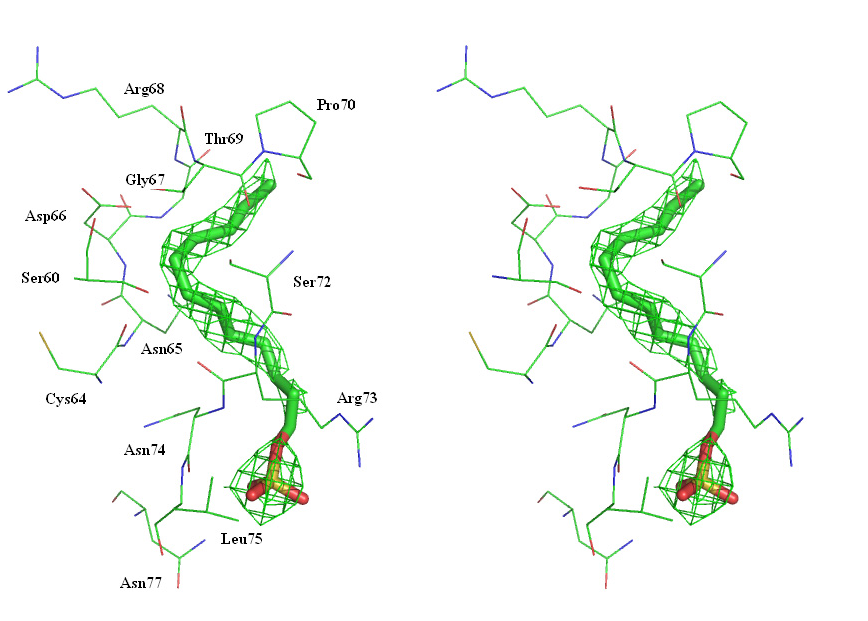
**Stereoview of the complex between SDS and the enzyme.** Electron density of the *2F*_*O*_-*F*_*C *_map is contoured at 1σ level for SDS. Figures 1 to 4 were generated by using PyMOL [17].

With the condition I buffer, as expected, no electron density was observed for MPD whereas a SDS molecule was found on the surface of lysozyme, exposed to the solvent. As suggested by Weiss et al, MPD may only be able to bind lysozyme at high pH values and/or at high concentration. SDS is loosely bound, as indicated by its temperature factors (70–80 Å for the sulphate tail). The SDS sulphate is salt bridged to Arg73 (Fig. 4) and the hydrocarbon tail makes contacts with Ser60, Cys64-Pro70, Ser72-Asn74. The binding position is different from those described in the SDS-lysozyme complex described earlier where the SDS molecules bind deeply into the protein [5]. Indeed, the hydrophobic interactions of SDS with the core of the protein are assumed to be lowered in the presence of MPD. In addition, the binding of SDS does not alter the structure of lysozyme. In this structure, no sodium or chloride ions were observed. It is worth noting that this study presents one of the very few structures of proteins co-crystallized with a denaturing concentration of SDS in its native form.

## Conclusion

This study, where crystals of lysozyme were grown for the first time in the presence of the amphipathic solvent MPD and denaturing concentrations of SDS, clearly established the preservation of the enzyme structure in a SDS/MPD mixture. It is hypothesized that high concentrations of MPD, changing the properties of SDS, would lower (condition I) or avoid (condition II) interactions between SDS and the protein. Indeed, in the form II crystal, even though it contains higher SDS concentration, but also higher MPD concentration, than in the form I, no SDS molecule was observed. This assumption is in agreement with the size-exclusion chromatography study described earlier [4], demonstrating that the protein no longer interacts strongly with SDS in a MPD buffer system. These structural data therefore support the hypothesis that MPD can protect proteins from SDS denaturation.

In addition, it is worth noting that this contribution shows one of the very few structures of proteins, in its native form, co-crystallized with SDS.

## Methods

### Crystallization

Lyophilized hen egg-white lysozyme, obtained from Sigma, was solubilized in 4 mM SDS, 50 mM Tris pH8, 150 mM NaCl and 2 M MPD (racemic form) (around 10 mg/ml protein). Crystals were grown at room temperature by the hanging drop vapor diffusion method using two different conditions. First 4 μl of the protein solution was mixed with 4 μl of a MPD-free reservoir solution containing 100 mM sodium acetate buffer pH 4.6 and 2 M sodium formate (condition I), and equilibrated with 1 mL of reservoir solution. In the second condition, the reservoir solution consists of 50 mM Tris pH8, 70% (~4.6 M) MPD (racemic form) and 6 mM SDS (condition II).

### Data collection, processing and refinement

Crystals harvested directly from mother liquor were flash-frozen in a 100 K nitrogen stream with no added cryoprotectant. Diffraction data of the form I crystals were collected using a Bruker MICROSTAR generator with a Bruker Proteum X8 CCD X-ray detector. The crystal-to-detector distance was 60 mm; 180 images (0.5° as oscillation range/image) with a 120 sec exposure time per image were collected; SAINT-Plus/Proteum was used to process the data.

For the form II crystals, a Gemini Ultra R system (4-circle kappa platform, Ultra Enhanced Cu Source, Ruby CCD detector) was used. The crystal-to-detector distance was 50 mm; 180 images with a 90 sec exposure time per image were collected; CrysAlis CCD and CrysAlis RED were used to process the data.

A molecular replacement solution was found using Phaser [11] with the molecular model of the native lysozyme (PDB entry 1Z55). Refinement was performed either with the Shelxl97 (Form II) [12] or Refmac5 (Form I) program [13]. Electron density maps were inspected with the graphic program Xtalview (Form II) [14] or Coot (Form I) [15], and the quality of the model was analyzed with the program Procheck [16]. The atomic coordinates and structure factors have been deposited in the Protein Data Bank (**3B6L**and **3B72**).

## Authors' contributions

CM carried out the crystallographic studies and wrote the manuscript. JP participated in the crystallogenesis experiments. GGP conceived of the study, and participated in its design and coordination and helped to draft the manuscript. JW helped in the study coordination and in the corrections of the paper. All authors read and approved the final manuscript.
